# Risk Factors of Cholera Transmission in Al Hudaydah, Yemen: Case-Control Study

**DOI:** 10.2196/27627

**Published:** 2021-07-05

**Authors:** Abdulqawi Mohammed Qaserah, Mohammed Abdullah Al Amad, Abdulwahed Abduljabbar Al Serouri, Yousef Saleh Khader

**Affiliations:** 1 Rapid Response Team Yemen Ministry of Public Health and Population Sana'a Yemen; 2 Yemen Field Epidemiology Training Program Yemen Ministry of Public Health and Population Sana'a Yemen; 3 Department of Community Medicine, Public Health, and Family Medicine Faculty of Medicine Jordan University of Science & Technology Amman Jordan

**Keywords:** cholera, outbreak, risk factors, Yemen, Field Epidemiology Training Program

## Abstract

**Background:**

Yemen has recently faced the largest cholera outbreak in the world, which started at the end of 2016. By the end of 2017, the cumulative reported cases from all governorates reached 777,229 with 2134 deaths. Al Hudaydah was one of the most strongly affected areas, with 88,741 (18%) cases and 244 (12%) deaths reported.

**Objective:**

The aim of this study was to determine the risk factors associated with cholera transmission in Al Hudaydah city, Yemen.

**Methods:**

From December 1, 2017 to January 10, 2018, a total of 104 patients with cholera (57 women and 47 men) who presented at cholera treatment centers in Al Hudaydah city with three or more watery stools in a 24-hour period and with moderate or severe dehydration were identified for inclusion in this study. Each case was matched by age and gender with two controls who were living in the neighboring house. A semistructured questionnaire was used to collect data on behavioral and environmental risk factors such as drinking water from public wells, storing water in containers, consumption of unwashed vegetables or fruits, and sharing a toilet.

**Results:**

The median age of the cases and controls was 20 years (range 5-80) and 23 years (range 5-85), respectively. Only 6% of cases and 4% of controls were employed. Multivariate analysis showed that eating unwashed vegetables or fruits (odds ratio [OR] 7.0, 95% CI 1.6-30.6, *P*=.01), storing water in containers (OR 3.0, 95% CI 1.3-7.3, *P*=.01), drinking water from a public well (OR 2.5, 95% CI 1.1-5.7, *P*=.02), and using a public toilet (OR 5.2, 95% CI 1.1-24.4, *P*=.04) were significantly associated with cholera infection risk.

**Conclusions:**

The cholera transmission risk factors in Al Hudaydah city were related to water and sanitation hygiene. Therefore, increasing awareness of the population on the importance of water chlorination, and washing fruits and vegetables through a health education campaign is strongly recommended.

## Introduction

Cholera is an extremely virulent disease that can cause severe acute watery diarrhea. Cholera is mainly transmitted by ingestion of food or water contaminated with the bacterium *Vibrio cholera* O1 or O139, and is considered to be a serious threat to global public health. An estimated 3-5 million cases and over 100,000 deaths of cholera occur each year worldwide [1]. The infection is often mild or without symptoms but can be severe and can kill the host within hours in some cases if left untreated [2]. This disease particularly spreads in countries where people have unfavorable living conditions such as poor access to safe water and sanitary toilets [1,2].

Drinking contaminated water and poor food preservation methods are the major risk factors for cholera transmission [3,4]. Different risk factors have been reported in previous research, such as bathing in the river, eating dried fish, not boiling drinking water, living with people who had acute diarrhea, travel and eating outside the home, and consumption of unrefrigerated leftover food [5-9].

Yemen has faced the largest cholera outbreak in the world in recent years [10,11]. The first cases appeared in late September 2016, and by the end of 2017, the cumulative reported cases from all governorates reached 777,229 with 2134 deaths. Al Hudaydah was one of the most strongly affected areas, with 88,741 (18%) cases and 244 (12%) deaths reported [12]. A recent study performed in Aden identified the following risk factors of cholera transmission: a history of travelling and having visitors from outside the Aden governorate; eating outside the house; not washing fruits, vegetables, and khat (a local herbal stimulant) before consumption; using common-source water; and not using chlorine or soap in the household [10]. Despite the fact that Al Hudaydah was one of the most strongly affected areas, the possible risk factors of transmission in this city are still unclear. Therefore, the aim of this study was to determine the risk factors associated with cholera transmission in Al Hudaydah city, Yemen.

## Methods

### Study Area and Setting

Hudaydah city is the center of Al Hudaydah governorate located on the coast of the Red Sea, which is the fourth largest city in Yemen with a population of 400,000 habitants. Al Hudaydah is also well known as a city in which the majority of the population live below the poverty line, and lacks public services such as water and sanitation. There are three main districts in Al Hudaydah: Al Meina, Al Hawak, and Al Hali. In response to the 2017 cholera outbreak, the World Health Organization launched five cholera treatment centers (CTCs), including three centers in the three general hospitals: Al Thawrah, Al Salakhanh, and Al Aulfi hospitals.

We performed a case-control study matched by age and gender. Cases included individuals who presented to CTCs in Al Hudaydah city with acute watery diarrhea (ie, three or more watery stools in a 24-hour period) and moderate or severe dehydration during the study period. Cases were included if they were at least 5 years of age, had a positive result to a rapid diagnostic test for cholera, and agreed to participate in the study. Cases were excluded if they lived outside of Al Hudaydah city after the start of the outbreak. Controls were selected from the same neighborhood of the cases among houses that had not reported any cases of cholera since the start of the outbreak. Individuals who were at least 5 years of age; had lived in the same neighborhood as the cases since the start of the outbreak up to April 27, 2017; did not have three liquid watery stools within 24 hours at any time since the start of outbreak; and agreed to participate in the study were included. Controls were specifically selected among individuals living in the house to the direct left of a case’s house. If a control was not found in that house, the data collectors moved to the next house on the left. Controls were excluded if they lived outside of Al Hudaydah city at any time after the start of the outbreak.

Cholera risk factors were defined as any event or behavior related to water and food consumption, and hygiene practices of peoples living in Al Hudaydah city that could potentially increase the chance of becoming infected with cholera.

### Sample Size

The sample size was calculated assuming that 40% of the controls had been exposed. To detect an odds ratio (OR) of 2 between any of the studied exposure factors and the disease with a margin of error of 5%, the minimum sample size was estimated as 104 cases and 208 controls (using a ratio of cases to controls of 1:2) at a level of significance of .05 and power of 80%. A total of 104 cases were recruited from the five CTCs in Al Hudaydah city from December 1, 2017 to January 10, 2018.

### Data Collection

Data were collected during the period from December 1, 2017 to January 10, 2018. Well-trained health workers collected data using a semistructured questionnaire. The questionnaire was translated to Arabic and distributed to 10 health care providers who were included in our pilot test. The questionnaire was modified accordingly, and the final version was used to collect responses through face-to face-interviews with the cases and controls. The interviewers collected the data from cases or their caretakers at CTCs after they reviewed the registers for admitted patients. Houses of cases were also visited to collect data related to water, sanitation, hygiene, and food consumed. The interviewers searched for controls at neighboring houses and selected two controls for each case.

The questionnaires were used to collect demographic characteristics such as age, gender, address, neighborhood, street, and occupation. Clinical details, including the date of diarrhea onset, symptoms, and diagnosis, were recorded. Information on travel history, contact with infected persons, hygiene practices, eating outside the home, and attending gatherings were also collected. Source of water in the home (eg, public well, truck water, private well/borehole water, water containers); water used for drinking, preparing food, and washing; as well as the source of food were assessed.

### Ethics

This study was performed as one of the requirements for graduation from the Yemen Field Epidemiology Training Program. Ethical approval was obtained from the ethical committee at Yemen Ministry of Public Health and Population. Verbal consent was obtained from each participant. Participation was strictly voluntary, and confidentiality of participants was maintained throughout the study.

### Data Analysis

Data were entered into Epi Info version 7.2. Data were summarized using frequency distributions. Percentages were compared using the OR. Univariate and multivariate binary logistic regression were used to determine factors associated with cholera. ORs with 95% CIs were calculated; a *P* value <.05 was considered statistically significant.

## Results

### Participant Characteristics

A total of 104 cases and 208 controls were included in this study. The median age was 20 and 23 years in the cases and controls, respectively. Only 6% of cases and 4% of controls were employed. Table 1 shows the demographic characteristics of the study participants.

**Table 1:** Demographic characteristics of study participants in Al Hudaydah, Yemen.

Characteristics		Cases (n=104)	Controls (n=208)
**Area of residence, n (%)**			
	Al Hali	81 (77.9)	162 (77.9)
	Al Hawak	15 (14.4)	30 (14.4)
	Al Meina	8 (7.7)	16 (7.7)
**Gender, n (%)**			
	Female	57 (54.8)	114 (54.8)
	Male	47 (45.2)	94 (45.2)
Age (years), median (range)		20 (5-80)	23 (5-85)
**Occupation, n (%)**			
	Employed	6 (5.8)	9 (4.3)
	Unemployed	53 (51.0)	117 (56.3)
	Student	45 (43.3)	82 (39.4)

### Factors Associated with Transmission

Table 2 shows the distribution of cholera risk factors among cases and controls in Al Hudaydah city, Yemen in 2017. A public well was more likely to be reported as a source of drinking water by cases compared to the controls. Cases were more likely than controls to use water containers to store water. Eating unwashed vegetables or fruits 1 week before the onset of symptoms was reported by significantly more cases than controls. Buying water products in sachets in the street in the last 7 days was also reported by significantly more cases than controls.

Compared with controls, cases also significantly reported more contact with another infected family member, traveling 1 week before the onset of symptoms, using a public toilet or pit latrine, and lack of tap water in the toilet. None of the other factors investigated was significantly associated with cholera infection.

**Table 2:** Univariate analyses of cholera risk factors reported among cases and controls.

Risk factors		Cases (n=104), n (%)	Controls (n=208), n (%)	OR^a^ (95% CI)	*P* value
**Source of home drinking water**				2.1 (1.2-3.7)	.008
	Public well	84 (80.8)	138 (66.3)		
	Truck	20 (18.3)	70 (33.7)		
Storing water in containers		92 (88.5)	149 (71.6)	3.0 (1.5-6.0)	.006
Eating unwashed vegetables or fruits		11 (10.6)	6 (2.9)	3.9 (1.4-11.0)	.007
Bought water or water products in sachets from the street in the last 7 days		58 (55.8)	83 (39.9)	1.7 (1.2-2.8)	.008
Contact with an infected person in the family		54 (51.9)	67 (32.2)	3.0 (0.5-18.6)	<.001
Travel history in the last 7 days		6 (5.8)	1 (0.5)	12.6 (1.5-106.7)	.002
**Type of toilet used**				2.7 (1.6-6.3)	.02
	Public	97 (93.3)	174 (83.7)		
	Private	7 (6.7)	34 (16.3)		
**Type of latrine used**				1.9 (1.2-3.2)	.005
	Pit latrine	63 (60.6)	91 (43.8)		
	Clean indoor latrine	41 (39.4)	117 (56.3)		
**Tap water in toilet**				1.9 (1.2-3.1)	.006
	No	61 (58.7)	88 (42.3)		
	Yes	43 (41.3)	120 (57.7)		

^a^OR: odds ratio.

### Multivariate Analysis of Risk Factors

The only factors that remained significantly associated with cholera in the multivariate analysis included eating unwashed vegetables or fruits, storing water in containers, drinking water from a public well, and using a public toilet (Table 3).

**Table 3:** Multivariate analysis for cholera transmission risk factors.

Risk factor	aOR^a^ (95% CI)	*P* value
Eating unwashed vegetables or fruits (yes vs no)	7.0 (1.6-30.6)	.01
Storing water in containers (yes vs no)	3.0 (1.3-7.3)	.01
Source of drinking water (public well vs truck)	2.5 (1.1-5.7)	.02
Type of toilet (public vs private)	5.2 (1.1-24.4)	.04

^a^aOR: adjusted odds ratio.

## Discussion

Cholera remains a global threat to public health, and an indicator of inequity and lack of social development [1]. Our study revealed that most of the cholera cases in Al Hudaydah, Yemen were in the Al Hali district, which is considered to be the poorest district in the city. This finding is in agreement with a study performed in an urban north-central Nigerian community in 2014 [3].

Drinking water from public wells was significantly associated with an increased odds of becoming infected with cholera. Two studies in Iran and Nigeria reported similar findings [9,13]. Storing water in containers is usually associated with a higher level of bacterial contamination if the water is not treated [13]. In our study, storing water in containers was associated with a three-fold increase in the odds of catching cholera. This finding is in agreement with two previous studies in Vietnam and Tanzania, respectively [5,7]. However, a study performed in urban slums showed that storing water in narrow-necked earthenware vessels (called a “sorai”) was effective in reducing the transmission of infection [14]. The differences between studies could be explained by the type of container used and likely also differences in the practice of treating water.

Eating unwashed vegetables or fruits was also significantly associated with an increased risk of cholera transmission, as reported in a previous study performed in Yemen [15]. Studies in Aden, South Sudan, and Nigeria reported the same finding [5,10,13]. Some studies reported that contact with a person having diarrhea and the presence of a cholera case at home are significantly associated with cholera transmission [13,14,16,17]. However, we did not find evidence to support these associations. Sharing a toilet was also a significant predictor of cholera transmission in this study and other studies [5].

The main limitation of this study is that it was performed in Al Hudaydah city and not in all governorates, and therefore the results cannot be generalized for the whole country.

In conclusion, the cholera transmission risk factors in Al Hudaydah city, Yemen were mainly related to water and sanitation hygiene. Increasing public awareness on the importance of daily water chlorination, and washing fruits and vegetables prior to consumption through a health education campaign is strongly recommended.

## Abbreviations

CTC: cholera treatment center
OR: odds ratio
